# Severe acute respiratory syndrome and COVID-19 under the hierarchy of the urban network of municipalities in the state of Acre, western Brazilian Amazon region, 2020-2021: a cross-sectional study

**DOI:** 10.1590/1516-3180.2021.0711.R1.20122021

**Published:** 2022-08-01

**Authors:** Mário Ribeiro Alves, Erlei Cassiano Keppeler

**Affiliations:** I PhD. Geographer and Associate Professor, Instituto de Saúde Coletiva (ISC), Universidade Federal de Mato Grosso (UFMT), Cuiabá (MT), Brazil.; II PhD. Biologist and Assistant Professor, Centro Multidisciplinar, Universidade Federal do Acre (UFAC), Rio Branco (AC), Brazil.

**Keywords:** Coronavirus, COVID-19, Pandemics, Amazon region, Municipalities, Urbanization

## Abstract

**BACKGROUND:**

The Respiratory Syndromes Surveillance System was created by the Brazilian Ministry of Health in 2000 to monitor influenza in this country. With the emergence of the new coronavirus pandemic, it became incorporated into the surveillance network for influenza and other respiratory viruses.

**OBJECTIVE:**

To analyze the transmission of coronavirus disease 2019 (COVID-19) and severe acute respiratory syndrome (SARS) in the state of Acre through its hierarchical urban network.

**DESIGN AND SETTING:**

Cross-sectional, descriptive and ecological study, using a spatiotemporal approach and using secondary data. This study was conducted in the state of Acre, northern Brazil.

**METHODS:**

This study used secondary data, and epidemiological weeks and municipalities were taken to be the units of analysis. Incidence rates and kernel intensities were calculated for four study periods. Spatiotemporal analysis was performed using scan statistics to identify clusters of SARS cases and considering the population of each municipality.

**RESULTS:**

In general, it could be observed that there were higher kernel rates and intensities in municipalities located in the north and south of this state (i.e. its most populous municipalities).

**CONCLUSION:**

Priority areas for interventions to control transmission of COVID-19 were highlighted, with the aim of reducing the risks of transmission to more distant areas in the urban hierarchy of the state of Acre.

## INTRODUCTION

Coronaviruses (CoVs) belong to a taxonomic group of viruses that cause respiratory infections and can affect humans and other animals.^1^ Severe acute respiratory syndrome coronavirus 2 (SARS-CoV-2) is a virus belonging to the Coronaviridae family, characterized by the shape of a crown, with a particle size between 60 nm and 140 nm. It is composed mainly of internal genetic ribonucleic acid (RNA) material, external proteins and a lipid bilayer. As in other viruses, SARS-CoV-2 contains nucleocapsid (N) proteins that are responsible for camouflaging the RNA, the genetic material of the virus, which is replicated in infected cells, thereby increasing the level of infection.^2,3^

The preventive measures usually publicized by public health authorities and the media encompass the following personal recommendations: avoid crowding, keep one’s distance from other people, stay at home, only go to commercial establishments in cases of extreme need, adopt social isolation, use masks and gloves and adopt use of delivery service; and traffic between cities should be blocked.^2^ Such measures aim to control transmission, involving individual and collective hygiene and physical distance actions.^4^

The Respiratory Syndromes Surveillance System was created by the Brazilian Ministry of Health in 2000 to monitor influenza in this country. With the emergence of the new coronavirus pandemic, it became incorporated into the surveillance network for influenza and other respiratory viruses.^5^ Data from June 24, 2021, revealed that there had been 18,243,483 confirmed cases of coronavirus 2019 (COVID-19) in Brazil, among which 16,511,701 individuals had recovered. There had been 509,141 deaths, corresponding to a mortality rate of 242.3 per 100,000 inhabitants of Brazil; death had occurred in 2.8% of the cases. In the state of Acre, there had been 85,128 cases (incidence of 9,652.4 per 100,000 inhabitants) and 1,735 deaths, with a mortality rate of 196.7 per 100,000 inhabitants of this state.^6^

## OBJECTIVE

In this context, the aim of this study was to analyze the transmission of COVID-19 and severe acute respiratory syndrome (SARS) in the state of Acre, in the west of the Amazon region, according to the hierarchical urban network of its municipalities, given the significant increase in deaths from COVID-19. It was hypothesized that the geographical accessibility of the municipalities would be a factor in its transmission, since shorter distance from the state capital would tend to contribute to greater transmission.

## METHODS

**Figure 1.**
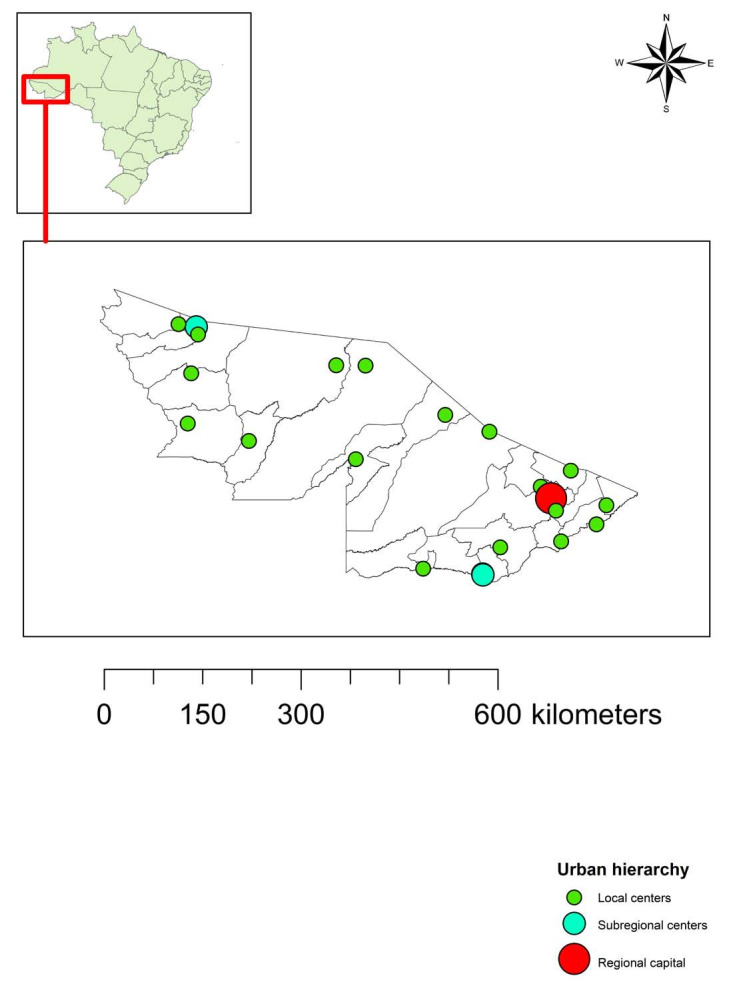
Location and hierarchy of municipalities in the state of Acre, Brazilian Amazon region.

This was a retrospective, descriptive and ecological study, using a spatiotemporal approach and using secondary data. Epidemiological weeks and municipalities were taken to be the units of analysis. This study was conducted in the state of Acre, northern Brazil, which had a projected population for the year 2021 of 906,876 inhabitants.^7^ Acre has 22 municipalities and the state capital is the municipality of Rio Branco (Figure 1).

The records of weekly cases of SARS for the period from January 8, 2020, to April 19, 2021, are freely accessible and were collected on April 23, 2021, from the Ministry of Health’s Respiratory Syndromes Surveillance System, which includes COVID-19 data.^5^ For the present study, the SARS case registry was used as a proxy (representative) of COVID-19 incidence, which had been justified through other studies using SARS data during the pandemic period.^8,9,10^ Furthermore, with the pandemic, the reinforcement of the SARS surveillance system represented an important step forward for public health, in the light of the epidemiology of respiratory agents.^11^

**Figure 2.**
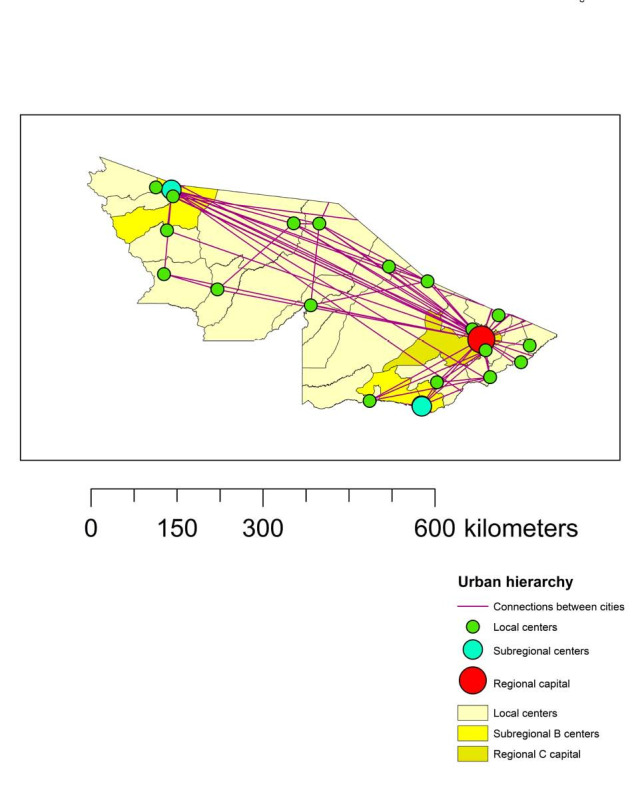
Accessibility and urban hierarchy of municipalities in the state of Acre, Brazilian Amazon region.

The Brazilian Institute for Geography and Statistics (Instituto Brasileiro de Geografia e Estatística, IBGE) was used as a source for data referring to the regions of influence (Regiões de Influência das Cidades, REGIC)^12,13^ and accessibility of the municipalities of Acre.^14^ The municipalities of this state are classified into three hierarchical levels: regional capital (Rio Branco), subregional centers (Brasiléia, Cruzeiro do Sul and Epitaciolândia) and local centers (other municipalities). In this hierarchical system, regional capitals are urban centers with a high concentration of management activities, but with a smaller reach of influence over the region than metropolises. Subregional centers are municipalities with less complex management activities, with a smaller extent of areas of influence than regional capitals (and the same is true for population size). At the last hierarchical level, local centers exert influence only within their territorial limits; they are able to attract residents from other cities in specific contexts, but are not the main destination from any other city. They have weak centrality of business and public management activities, and usually have links to other urban centers that are hierarchically higher with regard to purchasing/service activities and in relation to accessing public administration activities and business dynamics^12^ (Figure 2).

Estimated population data (2020) were obtained from the Ministry of Health.^15^ Digital grids for the municipalities and their respective main urban areas in the state of Acre were obtained from the IBGE.^16^

SARS data were filtered in terms of residents of the state of Acre and were then organized according to the municipalities of the state. Incidence rates and kernel intensities were calculated for four study periods, organized as the following ranges of reporting weeks: weeks 3 to 18, weeks 19 to 35 and weeks 36 to 53 of the year 2020, and weeks 1 to 12 of the year 2021.

The incidence rate was calculated by dividing the number of cases of SARS per period by the estimated population for each municipality, multiplied by 100,000. Kernel intensities are statistically calculated from incidence rate data and thus were also obtained by dividing the number of cases per period by the estimated population for each municipality, multiplied by 100,000. Nine intensity and adaptive radius strata were used.

Spatiotemporal analysis was performed using scan statistics to identify clusters of SARS cases and considering the population of each municipality. A Kulldorff statistical cylindrical scan was used, with a discrete Poisson probability distribution, to identify high-risk clusters by comparing the number of cases observed with the number of cases expected. The SaTScan software, version 9.6 (Kulldorff Information Management Services Inc., New York City, United States), was used for this.

Additionally, the relative risk (RR) was calculated for each cluster, based on the underlying population. The clusters were placed in order according to their log-likelihood ratio (LLR), such that the cluster with the highest LLR was the one that was least likely to have occurred by chance. The significant clusters (P-value < 0.05) had no geographical overlap and included a maximum of 50% of the city’s population.^17^ A circular radius of one kilometer was used, without geographical overlap.

From the municipal digital grid of Acre, a map of the incidence rates of SARS was made, along with a map of spatiotemporal clusters of SARS data and a map of accessibility and urban hierarchy, using the QGis software, version 2.18.20.^18^

Submission to and approval by an ethics committee was not necessary, given that the present study analyzed ecological data, without identifying the registered cases. These are open-access and freely available public secondary data.

## RESULTS

**Figure 3.**
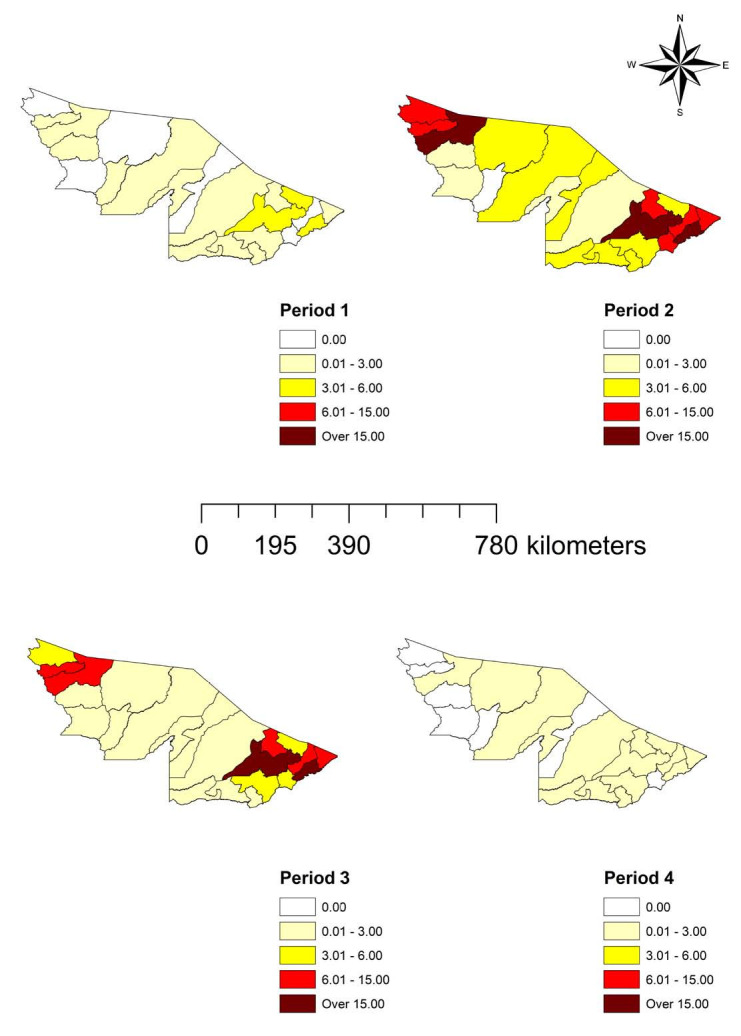
Severe acute respiratory syndrome rates per 100,000 inhabitants in the municipalities of the state of Acre, Brazilian Amazon region, 2020-2021.

From the analysis on the SARS rates, it could be observed that, in general, there were higher values in the municipalities located in the northern and southern parts of the state, especially in Cruzeiro do Sul, Rio Branco and Plácido de Castro. With lower values, the municipalities of Acrelândia, Bujari, Capixaba, Mâncio Lima, Rodrigues Alves and Senador Guiomard were highlighted. In the first study period, the highest rates of SARS were observed in Rio Branco (59.75), Plácido de Castro (35.08) and Porto Acre (31.87). In the second study period, the highest rates were observed in Plácido de Castro (451.01), Rio Branco (217.21) and Cruzeiro do Sul (163.91). During the third period, Plácido de Castro (275.62), Rio Branco (191.57) and Senador Guiomard (86.07) had the highest rates. In the last period, there was a reduction in these rates, such that all values werebelow 30.00: Bujari (28.79), Rio Branco (28.78), Epitaciolândia (21.39) and Xapuri (15.31) (Figure 3).

**Figure 4.**
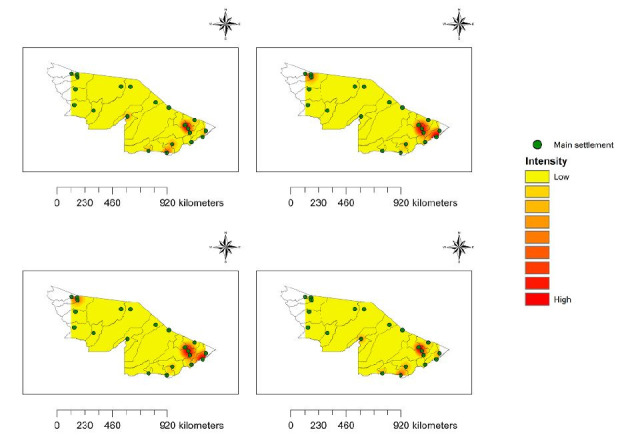
Kernel intensities of severe acute respiratory syndrome rates per 100,000 inhabitants in the municipalities of the state of Acre, Brazilian Amazon region, 2020-2021.

Regarding kernel intensities, in general, a spatial pattern similar to that of the SARS rates was observed, especially for the municipalities in the north and south of the state. In the first study period, the highest intensities were observed in the south of the state; among the municipalities with lower values, the kernel intensities in the municipalities of Assis Brasil, Jordão, Santa Rosa do Purus and Sena Madureira stood out. An increase in intensity was observed in the second period of the study, with concentration of higher values in the north and south of the state, and also in the municipalities of Brasiléia, Epitaciolândia, Feijó and Tarauacá. Similar patterns of intensity and spatial distribution were observed in the third period. In the last period of analysis, the municipalities in the south of the state were highlighted, along with the municipalities of Assis Brasil and Santa Rosa do Purus (Figure 4).

**Figure 5.**
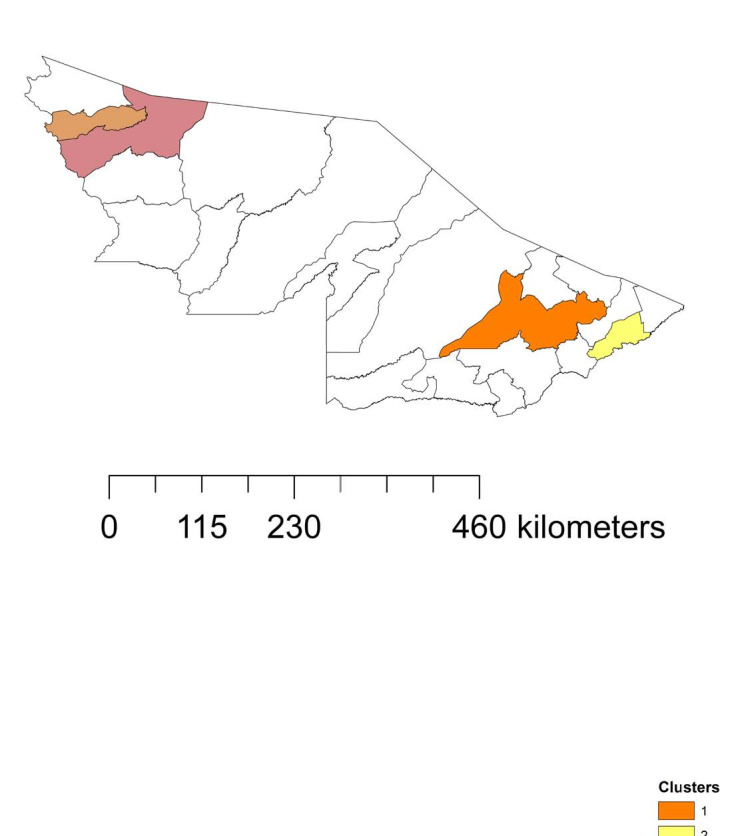
Spatiotemporal clusters of severe acute respiratory syndrome in municipalities in the state of Acre, Brazilian Amazon region, 2020-2021.

Initially, six spatiotemporal clusters were observed. However, two of these were not significant (P-value > 0.05) and were excluded. Each cluster was formed by only one municipality: cluster 1 (Rio Branco), cluster 2 (Plácido de Castro), cluster 3 (Cruzeiro do Sul) and cluster 4 (Rodrigues Alves). The highest LLR value was observed in cluster 1. The highest relative risk values were observed in clusters 2 (5.30) and 4 (4.26), and the same occurred in the relationship between observed cases and expected cases (5.10 and 4.24, respectively). Longer time durations were observed in clusters 1 and 2 (32 and 27 weeks, respectively) (Table 1 and Figure 5).

**Table 1.** Characteristics of spatiotemporal clusters of severe acute respiratory syndrome in municipalities in the state of Acre, Brazilian Amazon region, 2020-2021

Clusters	Duration (from first to last week)	Cases (observed/expected)	Relative risk	LLR	Number of municipalities
**Cluster 1**	17 to 48	2.39	4.10	667.75	1
**Cluster 2**	22 to 48	5.10	5.30	112.63	1
**Cluster 3**	21 to 39	2.07	2.14	37.49	1
**Cluster 4**	33 to 38	4.24	4.26	16.37	1

LLR = log-likelihood ratio.

## DISCUSSION

SARS-CoV-2 entered Brazil through the airspace, via passengers coming from Europe. Following the hierarchy of the Brazilian urban network, the disease spread through connections between airports. Later on, it spread through road transportation (which has different levels, ranging from interstate to commuting, using public transport), promoted through connections from hub cities to smaller municipalities within a given region of influence. Subsequently, transmission of the disease occurred through closer social relationships.^19^

The spatial distribution of COVID-19 cases has had a pattern similar to what was seen in other epidemics. It has shown relationships with intensities, frequencies, directions, duration and the qualitative characteristics of spatial interactions; and it has varied according to the stages of social relations, the population size and the technological standards of society (highlighting transportation itself). The international spread of the disease initially occurred through commercial passenger aviation, and this followed the configuration and logic of the urban hierarchy, air transportation and population arrangement of cities, such that its spread was faster and more spatially diffused in the most populous cities and capitals of countries and provinces.^19^

Those findings are corroborated through our results, considering that it was observed that cluster 1 (concerning the state capital, Rio Branco) began on week 17. This was the first cluster to be formed and was also the one with the longest temporal duration (Table 1), which may be explained by the greater population and greater influence of this municipality in the urban hierarchy of Acre, given that this is a regional capital (Figure 1). Reinforcing our findings, the first three cases of COVID-19 in Acre were registered in Rio Branco: one was diagnosed in a private clinic and two in municipal emergency rooms.^20^

Given that transmission of the disease occurs via social contacts, groups with larger demographic dimensions would, over time, tend to be more affected.^21^ This notion was reinforced by the findings of our study, as high incidence rates were observed in more populous cities, notably Cruzeiro do Sul (89,072 inhabitants) and Rio Branco (413,418 inhabitants).^15^ This can be explained by the fact that higher population density increases the risk of transmission of contagious diseases, thus making isolation at home necessary, which may even contain the spread of the disease such that it is kept away from municipalities in which there are no case records yet.^22^ Thus, cities with greater demographic density that have capillary internal transportation networks favor spatial dissemination of COVID-19,^23^ which may occur through flights or even through other forms of transportation/travel such as by road or river.

From analysis of Figures1 and 2, it can be seen that, in general, there is low connectivity between the cities in the state of Acre. Connections between cities is concentrated along the BR-364 highway, which links the two most populous cities in the state (Cruzeiro do Sul and Rio Branco). In addition, there was a certain level of connectivity between the municipalities close to the state capital (Rio Branco), due to its influence as a regional capital (Figure 2). Rio Branco is a center for logistics and communication services, which allows it to function as a meeting place, thereby adding value and flow to production and resources of all kinds. Through being endowed with information and knowledge infrastructure, a regional capital integrates into broader networks (national and even global).^24^

In the expansion process, a central agglomeration brings relationships closer and starts to share functions with neighboring municipalities and non-agglomerated centers. These can become incorporated to the same unit within a radius of approximately 200 kilometers. In general, urban-regional arrangements operate as receiving and diffusing spaces for decisions and capital, with integration of state, national and international scopes, such that these are marked out as focuses of concentration (and thus becoming established as the main centers of the Brazilian urban network).^25^

This scenario will have contributed to the increase in the numbers of SARS cases observed over the study period (Figures3 and 4), given that cities with greater economic activity would tend to have greater movement of people, thus contributing to the circulation of the virus at home and at work. This can be further aggravated by movement of migrant workers, who are often in contact with people from different locations, thereby contributing to increased disease transmission.^26^ Thus, identification of people with SARS, and consequently ensuring their isolation, can prevent transmission to other individuals.^27^

Our results showed that clusters 2, 3 and 4 started to form in week 21 (i.e. after the formation of cluster 1). This suggests that the number of cases of SARS in the municipalities of Acre spread outwards from Rio Branco. In fact, cluster 2 could possibly have been influenced by its proximity to Rio Branco, as it had a time duration of 27 weeks, in addition to having the highest RR among all the clusters (Table 1). Furthermore, the presence of regional airports and their importance in the regions of influence where they are located partly explains the cases of COVID-19 in more isolated regions,^19^ such as Cruzeiro do Sul (a subregional center with an airport) (Figure 2). Reinforcing our findings, a structured compartmental model (in agreement with data observed in large countries such as Brazil) indicated that maintenance of a high number of COVID-19 cases in Brazil could be supported by the geographical spread of the disease, which would occur from the state capitals to the interior of the country.^28^

Nevertheless, there was little connection between the municipalities in the middle of the state (Jordão, Marechal Thaumaturgo, Porto Walter and Santa Rosa do Purus), which is a possible explanation for the lower rates/intensities of SARS in these cities (Figures3 and 4). This can be explained by the low circulation of people between these municipalities, which would tend to give rise to low circulation of the disease. Corroborating our results, a study showed that the spread of the virus across Brazilian territory occurred from spaces with greater density of relationships, such that the country’s economic organization modeled the direction, temporality and intensity of COVID-19 cases. Thus, the spread of the disease across the country took on zonal, reticular and spot geographical features.^29^

It is noteworthy that the low level of testing in this country would probably be an explanation for the reduction in rates and intensities observed in the last study period, since Brazil is one of the countries that has done least testing in the world; it even has a lower testing rate than Uruguay, Argentina and Chile.^30^

Diffusion of COVID-19 is a global public health problem that poses a challenge that needs to be faced today. It is thus necessary to take a fresh look at new problems.^31^ In view of this pandemic, governments around the world have adopted a variety of measures with the objective of reducing infections and deaths, and their consequences in economic terms.^32^ The spread of the disease is related to the territorial division of labor, as seen in urban networks, which present hierarchies between cities. It is necessary to combat the spatial diffusion of the disease both through reduction of infections and through treatment of patients.^19^

Although rarely considered in socioeconomic development planning, city networks transform the territory, such that cities need to be analyzed together, in order to observe their relationships with other cities. In this way, the network of cities is integrated in a territorial cross-section. As an organic system, this demands integrated attention in order to raise the quality of flows between centralities and to plan physical and virtual movements.^24^

Regarding possible limitations of this study, a secondary database with provisional and incomplete records was used, which may have led to underestimation of SARS cases in Acre or even to information bias. This type of bias may also have occurred when the municipality of residence was recorded, which was informed by the respondent. As this was an ecological study, we must also highlight possible confounding in the analyses that were carried out, which may have occurred through the characteristics of this design. Furthermore, studies using approaches at aggregated levels do not allow inferences at individual level (ecological bias).

## CONCLUSION

From the analysis on the flow maps and power centers of the municipalities, it is possible to project a fairer future with regard to occupation of the territory, with more equal distribution of opportunities and more environmentally appropriate activities.^24^ Based on the above, priority areas for interventions to control the transmission of COVID-19 were shown, with the aim of reducing the risks of transmission to more distant areas within the urban hierarchy of the state of Acre, through controlling human mobility.^33^

Although our results reinforce the evidence regarding the spread of the disease through airports, roads and rivers can also be highlighted as transmission routes, as they facilitate contact between people from different locations.
